# Antioxidant Activity of Sprouts Extracts Is Correlated with Their Anti-Obesity and Anti-Inflammatory Effects in High-Fat Diet-Fed Mice

**DOI:** 10.1155/2021/8367802

**Published:** 2021-02-16

**Authors:** Chung Shil Kwak, Mi-Ju Kim, Sunyeong Park, In Gyu Kim

**Affiliations:** ^1^Institute on Aging, Seoul National University College of Medicine, Seoul 03080, Republic of Korea; ^2^Dain Natural Co., Seoul 04788, Republic of Korea; ^3^Department of Biochemistry and Molecular Biology, Seoul National University College of Medicine, Seoul 03080, Republic of Korea

## Abstract

Obesity is closely associated with oxidative stress and chronic inflammation leading to related metabolic diseases. Some natural extracts or polyphenols reportedly possess anti-obesity and anti-inflammatory effects as well as antioxidant activity. In this study, we assessed the correlations between the antioxidant, anti-obesity, and anti-inflammatory activities of plant extracts with potent antioxidant activity in diet-induced obese mice. Sprouts of *Cedrela sinensis* (CS) and *Oenothera biennis* L. (OB) were selected as the most potent antioxidant plant based on analysis of *in vitro* antioxidant activity of the extracts of ten different edible plants. C57BL/6 mice were fed with a high-fat diet (HFD) and orally treated with 50% ethanol extract of CS or OB at 50 or 100 mg/kg body weight 5 days a week for 14 weeks. Body weight gain, weight of adipose tissue, adipocyte size, and levels of lipid metabolism, inflammation, and oxidative stress markers were investigated. The CS or OB extract reduced body weight gain, visceral adipose tissue weight, adipocyte size, and plasma leptin levels, and expressions of adipogenic genes (PPAR*γ* and fatty acid synthase) in the adipose tissue and liver of HFD-fed mice. Both extracts also reduced mRNA levels of pro-inflammatory cytokines (IL-6 and TNF-*α*) and oxidative stress-related genes (heme oxygenase- (HO-) 1 and p40^phox^). Body weight gain of mice was significantly correlated with visceral adipose tissue weight and adipocyte size. Body weight gain and adipocyte size were significantly correlated with plasma total cholesterol and 8-epi PGF2*α* levels, mRNA levels of leptin, HO-1, p40^phox^, and CD-11 in the adipose tissue, and mRNA levels of TNF-*α* in the adipose tissue and liver. These results suggest that the CS and OB extracts with potent antioxidant activity may inhibit fat deposition in adipose tissue and subsequent inflammation.

## 1. Introduction

Obesity increases the risk of various metabolic diseases including type-2 diabetes, hypertension, fatty liver, and cardiovascular diseases. Obesity-induced oxidative stress and inflammation may cause the development of obesity-associated metabolic diseases [1]. High fat consumption leads to hyperlipidemia and subsequent increase of fatty acid oxidation and cellular reactive oxygen species (ROS), which activates the nuclear factor kappa beta (NF-*κ*B) pathway and thus increases the secretion of pro-inflammatory cytokines in various tissues [2, 3]. Particularly, fat accumulation in adipocytes induces the expression of NF-kB-dependent adipokines, which elicit inflammation by recruiting macrophage and neutrophil. These immune cells further stimulate the production of ROS by activating several enzymes such as NADPH oxidase and xanthine oxidase. Excessive ROS induce oxidative damage of DNA, lipids, and proteins in cells, thereby leading to various diseases [4, 5].

Chronic and sterile low-grade inflammation is frequently observed in the adipose tissue, liver, and kidneys of obese humans and rodents [6, 7]. Previous reports have implicated the increased number of inflammatory molecules including tumor necrosis factor-alpha (TNF-*α*), interleukin (IL)-6, inducible nitric oxide synthase (iNOS), transforming growth factor-beta (TGF-*β*), monocyte chemoattractant protein- (MCP-) 1, and plasminogen activator inhibitor (PAI)-1 secreted from visceral adipose tissue in the pathological processes of vascular stiffness, insulin resistance, and plaque formation in an arterial wall of obese animals [8–11]. These findings suggest that antioxidant therapy may be useful for the prevention of obesity-induced chronic diseases. In this regard, the discovery of novel antioxidant agents with anti-obesity and anti-inflammatory effects is important.

Polyphenols, antioxidants and prebiotics from natural plants, and probiotics have recently been shown to be effective for the prevention of obesity and its related complications in animals or humans [12–14]. The extracts of plant leaves and berries are rich sources of natural antioxidant. These extracts and some phytochemicals can reduce lipid accumulation and ROS generation in adipocytes by downregulating gene expression involved in adipogenesis and lipogenesis and/or by upregulating lipolysis-related gene expression [15, 16]. Resveratrol supplementation can attenuate the increased inflammatory response and oxidative stress in the adipose tissue of high-fat diet- (HFD-) induced obese mice by suppressing NF-kB signaling and by stimulating sirtuin 1 expression [1]. Another report described a significantly positive correlation between ROS production and lipid accumulation in 3T3-L1 cells treated with several wild herb extracts [17]. Interestingly, whole plant extracts and mixtures of flavonoids were reported to have superior effects compared to each isolated phytochemical [15, 18].

Young leaves and sprouts of various plants are consumed as a food in Korea, especially in springtime. To assess the relationship among the antioxidant activity, anti-obesity activity, and anti-inflammatory activity of plants, we compared *in vitro* antioxidant activity of the extracts from the edible parts of ten different plants. We also examined the anti-obesity and/or anti-inflammatory effects of two extracts with higher antioxidant activity using HFD-fed mice.

## 2. Materials and Methods

### 2.1. Sample Preparation and Extraction

Dried young leaves, sprouts, or aerial parts of *Ipomoea batatas* (IB), *Boehmeria nivea* (L.) *Gaudich* (BN), *Morus alba* L. (MA), *Acanthopanax koreanum* (AK), *Cedrela sinensis* (CS), *Oenothera biennis* L. (OB), *Equisetum arvense* L. (EA), *Artemisia princeps* Pamp. Hara (AP), *Oenanthe javanica* (Blume) DC. (OJ), and *Glebionis coronaria* (GC) were purchased from commercial sources, TOJONGHERB or ONSKYFARM (Namyangju, Korea). To obtain the sample extract, 1 kg of pulverized sample was stirred in ten volumes of 50% ethanol (Samchun, Seoul, Korea) for 24 hours at room temperature and reflux-extracted for another 24 hours at 80°C. The supernatant was filtered, concentrated using a rotary vacuum evaporator (Eyela, Tokyo, Japan), and dried using an oven dryer (Daesan Machinery, Gyeongju, Korea) for 48 hours at 70°C. Finely powdered sample from each extract was stored at −20°C.

### 2.2. In Vitro Antioxidant Activity

*In vitro* antioxidant activity of sample extracts was evaluated by two different methods using ascorbic acid (AA) as a positive control. First, 2,2-diphenyl-1 picrylhydrazyl (DPPH) radical scavenging activity was determined as per methods described previously [19]. The sample concentration that lowered the concentration of the radicals by 50% (IC_50_) was calculated. Second, ferric reducing antioxidant power (FRAP) was measured as per previously described methods [20]. The sample concentration showing the equal reducing activity to 10 *µ*g AA/mL (EC_10AA_) was calculated.

### 2.3. Animals, Diets, and Treatment

Male C57BL/6 mice (5-week-old) were obtained from DBL Co. (Eumsung, Korea). After one week of acclimatization, the mice were allocated into seven groups (*n* = 7–8 mice per group). The mice were fed with a rodent low-fat diet (LFD; D12450 B, Research Diet, New Brunswick, NJ) containing 3.82 Kcal/g, fat 10% kcal, protein 20% kcal, and carbohydrate 70% kcal or with a rodent high-fat diet (HFD; D12451, Research diet) containing 4.7 Kcal/g, fat 45% kcal, protein 20% kcal, and carbohydrate 35% kcal ad libitum for 14 weeks. Lycopene beadlets (LY; Lycored, Branchburg, NJ) containing 5% natural lycopene extracted from tomato was the positive control. Mice fed with HFD were treated orally with the CS extract, OB extract, LY, or vehicle (saline) 5 days a week. The animal groups are as follows: (1) LFD control (LFC) : LFD + vehicle treatment, (2) HFD control (HFC) : HFD + vehicle treatment, (3) LY : HFD + treatment with 50 mg LY/kg body weight, (4) CS-L : HFD + treatment with 50 mg CS extract/kg body weight, (5) CS-H; HFD + treatment with 100 mg CS extract/kg body weight, (6) OB-L : HFD + treatment with 50 mg OB extract/kg body weight, and (7) OB-H : HFD + treatment with 100 mg OB extract/kg body weight.

The mice were kept in a temperature- and humidity-controlled room. During the experiment, body weight and diet intake of animals were measured regularly. Diet was replaced every other day. Food consumption weight of the animals in each cage was estimated by subtracting the remaining weight from the supplied weight. Food efficient ratio (FER) was calculated as the total body weight gain/total food intake  ×  100.

The mice were starved overnight on the last day of the experiment. Blood was collected from the venous orbital plexus in EDTA-coated tubes (BD, Canaan, CT) under the inhalation anesthesia using 2.5% isoflurane (Sigma Aldrich, Saint Louis, MO) in oxygen. After cervical dislocation, liver and visceral adipose tissues (epididymal and perirenal) were removed and weighed. Some pieces of liver and epididymal fat tissue were frozen in Trizol reagent (QIAGEN, Hilden, Germany) or liquid nitrogen, and another piece of epididymal fat tissue was fixed in 10% paraformaldehyde solution. The experimental protocol was approved by the Institutional Animal Care and Use Committees of Woojung Genome Research Center (Suwon, Korea) (WJLACUC20191118-4-18).

### 2.4. Blood Biochemical Analysis

Concentrations of triglyceride and total cholesterol in plasma were determined using the AceChem kit (Youngdong Pharm., Korea). Aspartate aminotransferase (AST) and alanine aminotransferase (ALT) levels were measured using a commercial kit (Asan Pharm, Whasung, Korea). Malondialdehyde (MDA), 8-epi-prostaglandin (PG) F2*α*, and leptin levels in plasma were determined using commercial ELISA kit (Elabscience, Wuhan, China, or Komabiotech, Seoul, Korea) according to the manufacturer's instruction.

### 2.5. Measurement of Adipocyte Size of Mice

Fixed epididymal adipose tissues were processed routinely for paraffin embedding, sectioned, and then stained with hematoxylin-eosin (H&E). The morphology was observed at three fields a sample (x200) and the images were documented using an optical microscope with digital camera (Olympus, Tokyo, Japan). The size of adipocyte was analyzed from 60 adipocytes a sample (20 each field) using the Image J (NIH, Bethesda, MD).

### 2.6. Total RNA Extraction and Quantitative PCR (qPCR) Analysis

Total RNA of epididymal adipose tissue and liver were isolated using the RNeasy Lipid Mini kit (QIAGEN), and cDNA was synthesized using Superscript^®^II Reverse Transcriptase kit (Thermo Fischer Scientific, Waltham, MA). The mRNA expression levels of genes were analyzed using a Real-Time PCR system (Bio-Rad, Hercules, CA) and the SYBR FAST qPCR Master Mix (Kapa Biosystems, Wilmington, MA). Glyceraldehyde 3-phosphate dehydrogenase (GAPDH) was used as a reference gene. Relative gene expression levels were calculated using the 2^−ΔΔ^Ct method. The sequence of the primers used in this study is shown in Table 1.

### 2.7. Measurement of Thiobarbituric Acid-Reactive Substance (TBARS) Levels and Antioxidant Enzymes Activities in the Liver Tissue of Mice

To determine the TBARS concentrations in the liver, the liver sample was homogenized in 10 volumes of buffer containing 150 mM KCl, 50 mM Tris-HCl, and 1 mM EDTA (pH 7.4) using the Ultra-Turrax tissue homogenizer (IKA Works, Wilmington, NC). The homogenates were centrifuged at 800 × *g* for 10 min at 4°C and the supernatant (10% homogenate) was collected. TBARS concentrations in the 10% homogenate were determined according to the methods previously described [21]. To measure the antioxidant enzymes activities, the 10% homogenate was further centrifuged at 10,000 × *g* for 15 min at 4°C and the supernatant was collected. Previously described methods were used to determine catalase activity [22], superoxide dismutase-1 (SOD-1) activity [23], glutathione peroxidase (GPx) activity [24], and glutathione S-transferase (GST) activity [25]. The protein concentration of tissue sample was determined using the Pierce protein assay kit (Thermo Fisher Scientific, Waltham, MA).

### 2.8. Statistical Analysis

Data are presented as the mean ± standard deviation (SD). Significant differences between groups were determined by ANOVA, followed by the Duncan's multiple range test using SAS v9.4 (SAS Institute, Cary, NC). Correlations between variables were determined using the Pearson correlation coefficient. Statistical significance was set at *P* < 0.05.

## 3. Results

### 3.1. Potent In Vitro Antioxidant Activities of CS and OB Extracts

To compare the antioxidant activity of samples, we evaluated the DPPH radical scavenging activity (IC_50_) and FRAP (EC_10AA_) of the extracts. Data on the *in vitro* antioxidant activity and extraction yield of the extracts are summarized in Table 2. The OB extract showed the highest DPPH radical scavenging activity, followed by the CS extract. The OB extract also showed the highest FRAP, followed by the extracts of AK and CS. These results indicated the potent antioxidant activities of the OB and CS extracts. These extracts were selected for the animal study to explore their anti-obesity and anti-inflammatory effects.

### 3.2. CS and OB Extracts Reduce the Body Weight, Visceral Fat, and FER in HFD-Fed Mice

**Figure 1:**
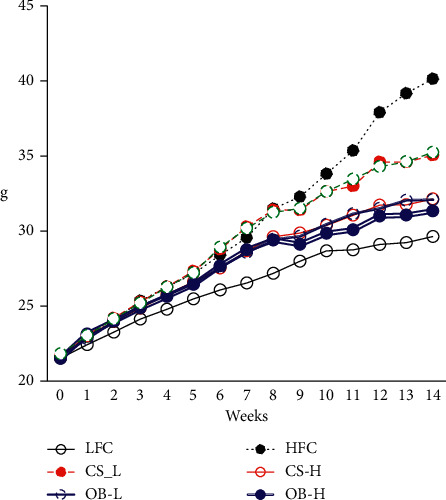
Effects of the CS and OB extracts on body weight of HFD-fed mice. C57BL/6 mice (five-week-old, male) were fed with low-fat diet (LFD) or high-fat diet (HFD) for 14 weeks. Mice were orally administered with lycopene beadlets (LY), CS, and OB extracts in saline at 50 mg/kg body weight (CS-L or OB-L) or 100 mg/kg body weight (CS–H or OB-H). Data are presented as the mean ± SD (*n* = 7–8/gr). 1) Means sharing the same alphabet are not significantly different at *P* < 0.05 as determined by ANOVA and Duncan's multiple range test. CS: *Cedrela sinensis*, OB: *Oenothera biennis* L.

The body weights of the HFC group were significantly higher than those of the LFC group after 4 weeks. The body weights of the OB-H group were significantly lower than those of the HFC group after 7 weeks. The body weights of the OB-L and CS-H groups and the CS-L and LY groups were significantly lower than those of the HFC group after 10 weeks and 13 weeks, respectively (Figure 1). At 14 weeks, body weight gains of the HFC group were increased approximately 2.3-fold compared with those of the LFC group. The average body weight gains of the LY, CS-L, CS-H, OB-L, and OB-H groups were reduced by 28.8%, 27.7%, 43.5%, 44.6%, and 46.7%, respectively, compared to the weight gains of the HFC group. These results indicate that supplementation with the CS and OB extracts, especially OB, reduced the body weight gains more effectively than LY treatment (Table 3).

The HFC group of mice consumed less diet but more energy than the LFC group. Treatment with the LY, CS, and OB extracts did not affect the food intake and energy intake of mice. Feeding the HFD resulted in an approximately 2.6-fold increase of FER compared with LFD feeding. The increase was significantly reduced by treatment with the LY, CS, and OB extracts. Particularly, the OB extract was more effective than the LY or CS extract (Table 3). Visceral fat weight measurements revealed that the HFD resulted in approximately 3.1-fold increase in epididymal and perirenal fat weight compared with the LFD. Consistently, treatment of HFD-fed mice with the LY, CS, or OB extracts significantly reduced the weight of visceral fat. Further, the average epididymal fat weight was reduced by 28.0%, 38.7%, 50.7%, 44.9%, and 51.2% in the LY, CS-L, CS-H, OB-L, and OB-H groups, respectively, compared with that of the HFC group. There is no significant difference in liver weights between the HFC and LFC groups. The average liver weight in the LFC group tended to be higher than that in the HFC group. Notably, liver weight was significantly reduced in CS-H, OB-L, and OB-H groups compared with the HFC group (Table 3). These results indicated the supplementation with the CS or OB extract inhibited fat accumulation, which was the main contributor to the reduced body weight gain in HFD-fed mice.

### 3.3. CS and OB Extracts Induce Reduction in Adipocyte Size and Subsequent Expression of Adipokines in the Adipose Tissue of HDF-Fed Mice

**Figure 2:**
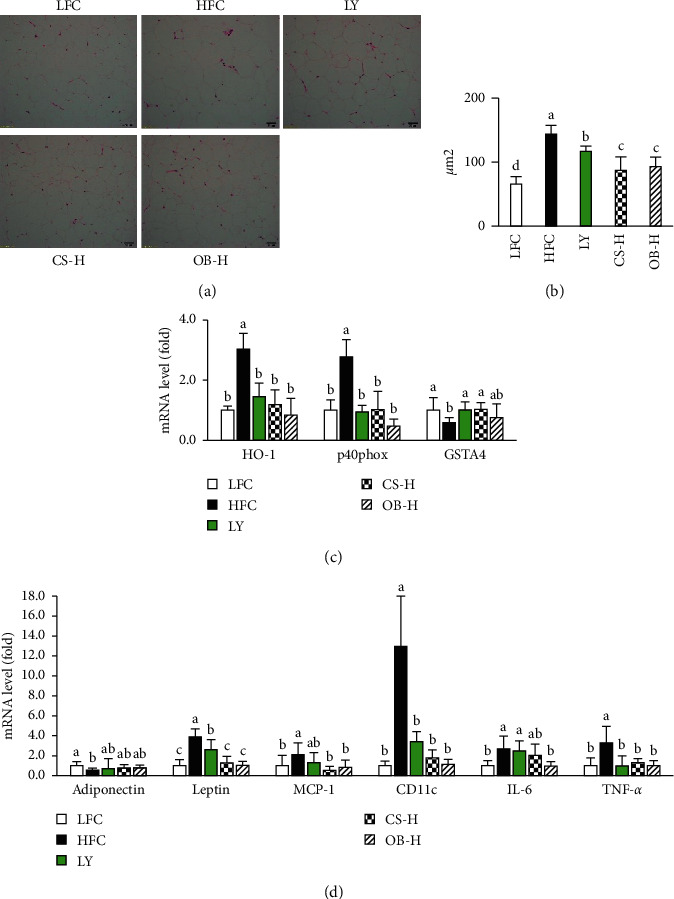
Effect of the CS and OB extracts on adipocyte size, expression of adipokines, and oxidative stress markers in epididymal adipose tissue of HDF-fed mice. Epididymal adipose tissues were stained with hematoxylin-eosin (H&E) (a) and size of adipocytes were measured by Image J (b). mRNA expression levels of oxidative stress-related markers (c) or inflammation markers (d) in epididymal adipose tissue were analyzed by quantitative RT-PCR (*n* = 5–6/gr). Means sharing the same alphabet in superscripts on the bars are not significantly different at *P* < 0.05 as determined by ANOVA and Duncan's multiple range test. HO-1: heme oxygenase-1, p40phox: NADPH oxidase subunit, GSTA4: glutathione S-transferase A4, MCP-1: monocyte chemoattractant protein, CD11c: a surface marker of M1 type macrophage, IL-6: interleukin-6, TNF-*α*: tumor-necrosis factor-*α*.

Increased adipocyte size is critical in obesity-related inflammatory states and is correlated with the production of adipocytokines [3]. To confirm the inhibitory role of the CS and OB extracts in the fat deposition at the cellular and molecular levels, we analyzed adipocyte size from the H&E-stained epididymal fat sections (Figure 2(a)). The adipocyte size in the HFC group was approximately 2.2-fold greater compared with the LFC group. The adipocyte sizes were significantly reduced in the LY, CS-H, and OB-H groups by 18.7%, 39.5%, and 35.4%, respectively, compared with that in HFC group (Figure 2(b)).

Treatment with the CS and OB extracts and LY significantly reduced mRNA levels of p40^phox^ and heme oxygenase-1 (HO-1), which are ROS generating enzymes, in adipose tissue of HFD-fed mice. In contrast, mRNA levels of GSTA4, detoxifying enzyme of lipid peroxidation products, were reduced significantly after HFD feeding. The mRNA levels were recovered by treatment with the LY or CS extract (Figure 2(c)).

We also investigated the expression levels of adipokines in epididymal fat. HFD feeding induced a significant increase of leptin, IL-6, and TNF-*α* mRNA levels and a decrease of adiponectin mRNA levels compared with LFC feeding. Treatment with the CS and OB extracts significantly reduced the mRNA levels of leptin, IL-6, and TNF-*α*, while it slightly increased adiponectin mRNA levels in HFD-fed mice. In contrast, LY treatment significantly reduced mRNA levels of leptin and TNF-*α*, but not IL-6 (Figure 2(d)). Adipokines induce inflammation in adipose tissue by recruiting and by activating immune cells [26]. To further confirm the effect of the CS and OB extracts, we analyzed the levels of MCP-1 and CD11c expression, which are markers for macrophage and leukocyte, respectively, in epididymal fat. HFD feeding resulted in a 2.1-fold increase in MCP-1 mRNA and a 12.9-fold increase in CD11c mRNA compared with LFD feeding. Treatment with the CS and OB extracts significantly reduced both levels, whereas LY treatment significantly reduced CD11c levels, but did not reduce MCP-1 levels (Figure 2(d)). These results demonstrated that supplementation with the CS or OB extract inhibited HFD-induced fat deposition and subsequent adipokine-induced inflammation more effectively than LY.

### 3.4. CS and OB Extracts Reduce Expression of Inflammatory Cytokines, Antioxidant Enzymes, and Lipid Peroxidation in the Liver of HDF-Fed Mice

**Figure 3:**
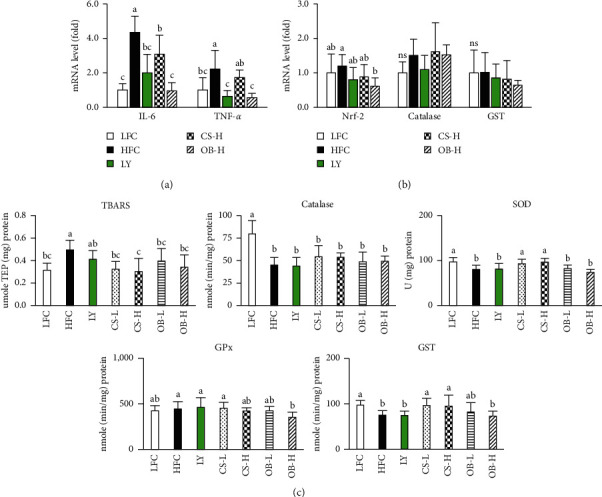
Effects of the CS and OB extracts on the expression levels of inflammatory cytokines, oxidative stress-related genes, and lipid peroxidation levels in the liver of HDF-fed mice. Expression levels of inflammatory cytokines (a) and oxidative stress markers in the liver (b) were analyzed by quantitative RT-PCR (*n* = 5-6/gr). Levels of lipid peroxidation and activities of antioxidant enzymes were measured (*n* = 7–8/gr) as described in Materials and Methods (c). Means sharing the same alphabet in superscripts on the bars are not significantly different at *P* < 0.05 as determined by ANOVA and Duncan's multiple range test. ns: not significant, Nrf-2: nuclear factor erythroid 2-related factor 2, GST: glutathione S-transferase, TBARS: thiobarbituric acid-reactive substance, SOD: superoxide dismutase, GPx: glutathione peroxidase.

Although no difference in liver weight was observed between the HFC and LFC group, treatment with the OB extract significantly reduced liver weight in HFD-fed mice (Table 3). Fatty liver leads to increased oxidative metabolism, which elevates hepatic oxidative stress and inflammatory responses [27]. We examined the levels of inflammatory cytokines and oxidative stress markers in the liver. mRNA levels of IL-6 and TNF-*α* in the liver of HFC group were increased approximately 4.3-fold and 2.2-fold compared with those in the LFC group. Treatment with the LY, CS, or OB extract resulted in significantly decreased expression of IL-6 and TNF-*α* in the liver of HFD-fed mice. The reductions attributed to the LY and OB extract were significantly higher than the CS extract (Figure 3(a)). Next, we evaluated the hepatic response to oxidative stress by measuring the mRNA expression levels of nuclear factor erythroid 2-related factor- (Nrf-) 2, catalase, and GST. HFD feeding had little effect on their mRNA levels, but treatment with the OB extract significantly reduced mRNA expression of Nrf-2, but not the expression of catalase and GST, in HFD-fed mice (Figure 3(b)). Additionally, we measured the levels of lipid peroxidation and antioxidant enzyme activity in the liver. The HFC group showed an approximate 1.6-fold increase of TBARS concentration compared with the LFC group. The increased hepatic TBARS levels led by HFD were significantly reduced by treatment with the CS and OB extracts, but were not reduced by LY treatment. Treatment with the OB extract significantly reduced GPx activity, whereas treatment with the CS extract increased SOD and GST activities (Figure 3(c)).

### 3.5. CS and OB Extracts Reduce Expression of Adipogenic Genes in the Adipose Tissue and Liver of HDF-Fed Mice

**Figure 4:**
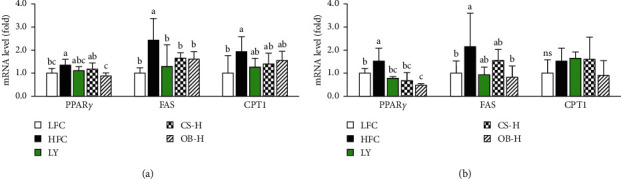
Effects of the CS and OB extracts on mRNA expression of lipid metabolism involved genes in the adipose tissue and liver of HDF-fed mice. Expression level of each gene in the adipose tissue (a) and liver (b) was analyzed by quantitative RT-PCR (*n* = 4–6/gr) as described in Materials and Methods. Means sharing the same alphabet in superscripts on the bars are not significantly different at *P* < 0.05 as determined by ANOVA and Duncan's multiple range test. PPAR*γ*: peroxisome proliferator activator receptor *γ*, FAS: fatty acid synthase, CPT-1: carnitine palmitoyl transferase-1, ns: not significant.

We analyzed mRNA expression of genes involved in adipogenesis or mitochondrial oxidation of fatty acids in epididymal adipose tissue and liver. As shown in Figure 4(a), HFD feeding significantly increased mRNA expression of peroxisome proliferator activator receptor gamma (PPAR*γ*), fatty acid synthase (FAS), and carnitine palmitoyl transferase-1 (CPT-1) in the adipose tissue compared with LFD feeding. HFD-induced FAS expression was significantly reduced by treatment with the CS and OB extracts and LY, while HFD-induced PPAR*γ* expression was significantly reduced by treatment with the OB extract. HFD also significantly increased mRNA expression of PPAR*γ* and FAS in the liver. HFD-induced PPAR*γ* expression in the liver was significantly reduced by treatment with the CS and OB extracts and LY, while HFD-induced FAS expression was significantly reduced only by the OB extract (Figure 4(b)). CPT-1 mRNA expression in the liver was not different among the groups. These results demonstrated that oral treatment with the CS or OB extract suppressed expression of adipogenic genes in adipose tissue and liver of HFD-fed mice, but did not influence significantly mitochondrial oxidation of fatty acids.

### 3.6. CS and OB Extracts Reduce Plasma Levels of Leptin and Oxidative Stress Markers

**Figure 5:**
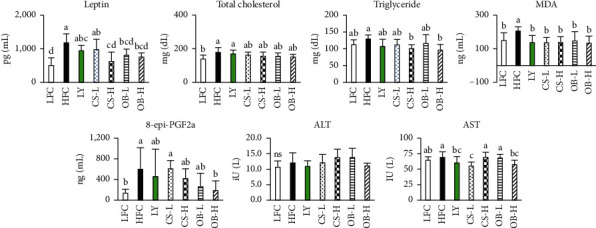
Effects of the CS and OB extracts on the levels of leptin, lipids, and oxidative stress markers in the plasma. Concentration of triglyceride, total cholesterol, and activity of aspartate aminotransferase (AST) and alanine aminotransferase (ALT) in plasma were determined using commercial kits. Malondialdehyde (MDA), 8-epi-prostaglandin (PG) F2*α*, and leptin levels were determined by ELISA kits according to the manufacturer's instruction. Means sharing the same alphabet in superscripts on the bars are not significantly different at *P* < 0.05 as determined by ANOVA and Duncan's multiple range test.

To evaluate the systematic effect of extracts in HFD-fed mice, we analyzed the plasma levels of adiposity, oxidative stress, and hepatic toxicity. HFD feeding resulted in a 2.3-fold increase of plasma leptin levels compared with LFC feeding. The increase was significantly reduced by 47.7% in the CS-H group, 31.5% in the OB-L group, and 35.4% in the OB-H group, but not in the CS-L and LY groups (Figure 5). Similarly, analysis of markers for oxidative stress revealed that HFD feeding produced a 1.4- and 4.5-fold increase of plasma MDA and 8-epi-PGF2*α*, respectively, compared with LFC feeding. Treatment with the LY, CS, or OB extract produced a 28.9% to 36.8% reduction in MDA levels compared with the HFC group. The OB-H group displayed significantly reduced 8-epi-PGF2*α* levels compared with the HFC group (Figure 5).

Plasma levels of triglycerides were significantly reduced in the CS-H and OB-H groups compared with the HFC group. The total cholesterol levels remained unaffected by any treatments. There was no significant difference in plasma AST and ALT levels between the LFC and HFC groups. The levels of AST, but not ALT, were significantly reduced in the CS-L and OB-H groups compared with the HFC group (Figure 5).

### 3.7. Correlations between Markers of Obesity and Inflammation or Oxidative Stress in Mice

To confirm the effect of the reduced body weight gains on the levels of markers for inflammation or oxidative stress in mice, we examined their statistical correlation. Body weight gain was significantly positively correlated with epididymal fat weight, adipocyte size, and plasma leptin level. Additionally, body weight gain was positively correlated with immune cell infiltration (CD11c), inflammatory cytokine levels (TNF-*α*, IL-6, and MCP-1) in the adipose tissue, IL-6 and TNF-*α* levels in liver, and markers for oxidative stress (plasma MDA and 8-epi-PGF_2_*α*, hepatic TBARS, and adipose HO-1, p40^phox^, and GSTA4) (Table 4). Adipocyte size was positively correlated with plasma leptin and 8-epi-PGF_2_*α* levels and leptin, HO-1, p40^phox^, GSTA4, TNF-*α*, and CD11c expressions in the epididymal fat. Body weight gain and adipocyte size were negatively correlated with antioxidant enzyme activities in the liver, such as catalase and SOD-1. These results demonstrate that the anti-obesity, antioxidant, and anti-inflammatory activities of CS and OB extracts in HFD-fed mice were mutually influenced.

## 4. Discussion

CS and OB belong to Meliaceae family and Onagraceae family, respectively. Both plants grow in Asian region, including Korea [26, 28]. The roots or all parts of OB have been used in Korea as a folk medicine to alleviate inflammation and fever. Ethanol extract of OB sprouts can scavenge DPPH radicals and inhibit the production of pro-inflammatory mediators in lipopolysaccharide- (LPS-) treated macrophages [29, 30]. Methanol extract of aerial parts of OB can inhibit ROS production in neutrophils and demonstrates anti-inflammatory activity via the suppression of hyaluronidase and lipoxygenase [31]. The representative compounds in the extract of OB aerial parts were reported to be a part of flavonoid glycosides, such as kampherol-3-O-glucoside, quercetin-3-O-galactoside, quercetine-3-O-rhamnoside and myricetin-3-O-glucoside, phenolic acids, and tannins [32].

CS has been also used as a folk medicine for treatment of enteritis, dysentery, and itching [26]. Ethanol extract of CS sprouts reportedly suppresses alcohol-induced oxidative stress in HepG2 cells by upregulating GPx and HO-1 expressions [33]. Dichloromethane extract of CS sprouts strongly inhibits ROS and nitric oxide production by downregulating iNOS and COX-2 in LPS-treated macrophages [34].

In this animal study, the treatment levels of the CS and OB extracts (50 mg/kg BW and 100 mg/kg BW) and LY (50 mg/kg BW) were settled based on the previous reports. Oral treatment with 1 mg lycopene/kg/day (corresponding to 20 mg LY/kg BW) to C57BL/6J mice inhibited HDF-induced adiposity and inflammatory response, but failed to reduce the body weight gain [35]. Kim et al. [36] observed that oral treatment (100 mg/kg BW) with the extract of *Toona sinensis* leaf (TS), which is similar to CS, attenuated inflammatory responses in LPS-injected mice. Oral treatment with the TS extract (40–300 mg/kg BW) exerted the antifatigue and antioxidant activities in various animal models [37–39]. Additively, treatment with the ethanol extract of Mulberry leaves or *Aralia elata* sprouts (50 mg/kg BW) resulted in reduction of body weight and fat weight in HFD-fed mice [40, 41] and modulation of activities of antioxidant enzymes in carcinogen-treated mice [42].

It is noteworthy that treatment with CS or OB extract to HFD-fed mice ameliorated the body weight gain and visceral fat accumulation without affecting food intake and energy intake in this study. Consistently, FER was significantly reduced in CS or OB extract treated group, especially more effectively in OB-L and OB-H group, compared to that in HFC group. The anti-obesity effect of CS and OB extract in HFD-fed mice might have resulted from suppressing the expression of adipogenic genes, such as PPAR*γ* and FAS, rather than stimulating metabolic oxidation of fatty acids as measured by CPT-1 expression in this study. PPAR*γ* is a transcriptional regulator that controls adipocyte proliferation, lipogenesis, and lipolysis. FAS functions as a key enzyme in de novo synthesis of fatty acid [43, 44]. CPT-1 is involved in mitochondrial oxidation of fatty acids via catalysis of the transfer of fatty acids from cytosolic compartment into mitochondrial compartment. Zhao et al. [13] reported that excessive oxidative stress might decrease energy expenditure in adipocytes through the induction of mitochondrial dysfunction and affect the expressions of genes and activities of enzymes involved in lipid metabolism.

On the oxidative stress markers, it was reported that adipose tissue of HFD-induced obese mice exhibited an increased expression of p40^phox^ and HO-1, and a decreased expression of GSTA4 and GSTA3 selectively [45]. The p40^phox^, a subunit of NADPH oxidase, is a major enzyme responsible for ROS generation in the macrophages and involved in generation of superoxide radical and H_2_O_2_ in adipocytes [46, 47]. HO-1, one of the target genes of Nrf-2, is upregulated in obese mice and a microsomal enzyme induced in response to oxidative stress and some cytokines and protects against oxidative stress, inflammation, and metabolic dysregulation [48, 49]. Nrf-2, an emerging regulator of cellular resistance to oxidants, is a critical transcription factor for regulating the expression of genes involved in antioxidant defense, oxidant signaling, and drug metabolism [11, 50]. GSTA4, an antioxidant enzyme detoxifying lipid aldehyde, catalyzes the glutathionylation of reactive *α*, *ß*-unsaturated aldehydes which covalently modify protein and DNA and activate cellular stress-response systems. It has been demonstrated that downregulation of GSTA4 in adipose tissue led to increased ROS production, protein carbonylation, and mitochondrial dysfunction contributing to the development of insulin resistance [45, 51].

In the present study, we demonstrated how the CS or OB treatment reduced oxidative stress induced by HFD. The CS and OB treatments reduced MDA levels in the plasma and TBARS levels in the liver, downregulated the expression of HO-1 and p40^phox^ in the adipose tissue, and modulated activity of antioxidant enzymes such as SOD, GPx, and GST in the liver of HFD-fed mice. In addition, the CS treatment increased GSTA4 expression in the adipose tissue, and the OB treatment reduced Nrf-2 expression in the liver.

There is a limitation in this study that we did not examine the activity of antioxidant enzymes or oxidative damage markers in the adipose tissue. However, we need to pay attention to the strong antioxidant property of the CS and OB extracts in diet-induced obese animals because the increased ROS production in obesity is known to be positively correlated with lipid accumulation [17].

We elucidated the correlation between antioxidant or anti-inflammatory markers and anti-obesity markers in mice in this study. Body weight gain was well correlated with the oxidative stress levels in the plasma and liver. The reduction of body weight gain was dose-dependent in the mice treated with the CS or OB extract. Additionally, body weight gain was significantly correlated with epididymal fat weight, adipocyte size, and plasma leptin, a marker for body adiposity, although the CS or OB extract had no effect on energy intake. These results indicate that antioxidant activity of the CS or OB extract probably results in the inhibition of fat accumulation in adipocytes.

Sprouts or buds of plants are enriched in nutrients, antioxidant vitamins, and phytochemicals, such as quercetin, epigallocatechin gallate, curcumin, anthocyanins, and resveratrol [28]. These polyphenols have been reported to alleviate oxidative stress, to reduce chronic low-grade inflammation, and to inhibit lipogenesis, thereby improving insulin resistance and reducing risk of cardiovascular diseases [3, 13, 49, 52, 53]. Methanol extract of aerial parts of OB contains high levels of gallic acid, ellagic acid, caffeic acids, quercetin, and kaempferol [54]. CS sprouts contain abundant limonoids, triterpenoids, and flavonoids, such as quercetin and quercitrin [33, 41, 55]. Lycopene is a carotenoid that exhibits antioxidant activity and a reducing effect on body weight and hepatosteatosis in obese animals [10, 12, 36]. Thus, we used a commercial lycopene supplementary product containing 5% tomato lycopene, 2% tocopherol, and 2.5% ascorbyl palmitate as a positive control in this study.

Our results suggest that polyphenols and flavonoids in the CS or OB extract may inhibit fat deposition in adipocytes, possibly via direct inhibition of key enzymes involved in transport of fatty acids from plasma lipoproteins to adipocytes, such as lipoprotein lipase in capillary wall, and/or stimulating synthesis of fatty acids and triacylglycerol by influencing FAS and glycerol-3-phosphate acyltransferase. Another possibility is that reduction in redox status conferred by their antioxidant activity inhibits transport and reesterification of fatty acids [56]. Further studies are needed to establish the role of redox homeostasis in the regulation of lipid deposition in adipocytes.

It has been reported that adipocytes secrete a number of bioactive proteins called adipokines, which include pro-inflammatory cytokines and leptin. Adipokine levels have been correlated with adipocyte size or adipose tissue mass [11, 57]. Increased leptin and resistin levels result in immune cell infiltration into the adipose tissue through the increase of vascular permeability, which subsequently elevates oxidative stress and inflammatory responses thereby leading to metabolic disorder [57]. Recruited macrophages and neutrophils and infiltrated macrophages in tissue are polarized into M1 cells which in turn copiously secrete pro-inflammatory cytokines and ROS, eventually leading to further amplification of inflammation, oxidative damage of cellular components, and activation of redox signaling pathways [9, 11, 58].

Moreover, we observed the correlations between the levels of obesity markers and the levels of inflammatory or oxidative stress markers are more prominent in the adipose tissue than those in the liver in this study. The expression levels of several pro-inflammatory and oxidative stress markers in the adipose tissue were significantly correlated with body weight gain. In the liver, however, only the expression levels of TNF-*α* and IL-6 were significantly correlated with body weight gain. In support of these results, a previous report showed that tissue inflammation occurred in adipose tissue, liver, kidneys, and intestine of diet-induced obese mice; however, visceral adipose tissue was more inflamed than the other tissues [8]. Another study revealed that adipose tissue inflammation was established prior to the development of hepatic inflammation. Therefore, adipose tissue inflammation would contribute more to the development of metabolic disorders compared to hepatic inflammation [6].

Taken together, our results suggest that decreases of inflammation and oxidative stress in HFD-fed mice due to the supplementation of the CS or OB extract may be attributed to the reduced adiposity and possibly direct antioxidant activity of the extract as well.

## 5. Conclusion

Oral treatment with 50% ethanol extracts with high antioxidant activity obtained from CS and OB sprouts significantly reduced body weight gain in HFD-fed mice, presumably via inhibition of fat deposition, and subsequent obesity-induced inflammatory response and oxidative stress. The anti-obesity and antioxidant activities of the CS and OB extracts might be resulting from the biological actions of phytochemicals present in the extracts. Our results suggest that the extract of plant sprouts with high antioxidant activity may potentially mitigate obesity and inflammation induced by a HFD, thereby preventing obesity-related metabolic diseases. Further research is needed to identify the effective components in the CS and OB extracts and to elucidate their functional mechanisms.

## Figures and Tables

**Table 1:** Sequence of primer for qPCR.

Gene		Forward	Reverse
Leptin	*LEP*	5′-TGT GCT GCA GAT AGC CAA TGA-3′	5′-TGG AGA AGG CCA GCA GAT G-3′
Adiponectin	*ADIPOQ*	5′-AAC CCC TGG CAG GAA AGG-3′	5′-TGA ACG CTG AGC GAT ACA CAT-3′
Catalase	*CAT*	5′-CAC ACC TAC ACG CAG GCC GG-3′	5′-CTG CGC TCC GGA GTG GGA GA-3′
Nuclear factor erythroid 2-related factor 2	*Nrf-2*	5′-AAG CTT TCA ACC CGA AGC AC-3′	5′-TTT CCG AGT CAC TGA ACC CA-3′
Glutathione S-transferase	*Gst*	5′-TGC AGA CCA AAG CCA TTC TC-3′	5′-ACG GTT CCT GGT TTG TTC CT-3′
Glutathione S-transferase A4	*GstA4*	5′-CGA TGG GAT GAT GCT GAC ACA-3′	5′-CAC TGG GAA GTA ACG GGT TTT AGC-3′
Heme oxygenase-1	*HO-1*	5′-CCT CAC TGG CAG GAA ATC ATC-3′	5′-CCT CGT GGA GAC GCT TTA CAT A-3′
Monocyte chemoattractant protein-1	*MCP-1*	5′-CCA GCA CCA GCA CCA GCC AA-3′	5′-TGG GGC GTT AAC TGC ATC TGG C-3′
NADPH oxygenase	*p40* ^*phox*^	5′-CCT GCC CAC ATT GCC AGC CA-3′	5′-AGA CCG GCA GGC TCA GGA GG-3′
M1 macrophage marker	*CD11c*	5′-CTG GAT AGC CTT TCT TCT GCT G-3′	5′-GCA CAC TGT GTC CGA ACT C-3′
Interleukin-6	*IL-6*	5′-CCA GAG ATA CAA AGA AAT GAT GG-3′	5′-ACT CCA GAA GAC CAG AGG AAA T-3′
Tumor-necrosis factor-*α*	*TNFα*	5′-CCA GAC CCT CAC TAG ATC A-3′	5′-CAC TTG GTG GTT TGC TAC GAC-3′
Peroxisome proliferator activator receptor *γ*	*PPARγ*	5′-GCC CAC CAA CTT CGG AAT C-3′	5′-TGC GAG TGG TCT TCC ATC AC-3′
Fatty acid synthase	*FAS*	5′-AGC CCA CGT CGT AGC AAA CCA-3′	5′-AGC CCA CGT CGT AGC AAA CCA-3′
Carnitine palmotoyl transferase-1	*CPT-1*	5′-TGT TGG GTA TGC TGT TCA TGA CA-3′	5′-GCG GCC TGG GTA GGA AGA-3′
Glycealdehyde-3-phosphate dehydrogenase	*GAPDH*	5′-GGG AAG CCC ATC ACC ATC T-3′	5′-CGG CCT CAC CCC ATT TG-3′

**Table 2:** Extraction yield of sample and *in vitro* antioxidant activities of the extract.

	Plant name	Local name	Sample	Extraction yield^1)^ (%)	DPPH^2)^ radical scavenging activity	FRAP^3)^
					IC_50_^4)^ (*µ*g/mL)	EC_AA10_^5)^ (*µ*g/mL)
	Ascorbic acid (AA)			—	2.35 ± 0.72^6)^	—
1	*Ipomoea batatas* (IB)	Goguma	Leaves	18.8	392.4 ± 53.7	165.5 ± 46.3
2	*Boehmeria nivea* (L.) *Gaudich* (BN)	Mosi	Leaves	31.8	375.9 ± 61.5	67.8 ± 6.3
3	*Morus alba* L. (MA)	Pong	Leaves	24.4	826.0 ± 26.2	195.3 ± 4.8
4	*Acanthopanax koreanum* (AK)	Sum-ogalpi	Leaves	29.4	61.1 ± 1.1	39.1 ± 1.3
5	*Cedrela sinensis* (CS)	Gazuk	Sprouts	20.1	35.9 ± 7.1	39.5 ± 2.5
6	*Oenothera biennis* L. (OB)	Dalmazi	Sprouts	25.4	26.2 ± 4.2	15.8 ± 1.1
7	*Equisetum arvense* L. (EA)	Shettgi	Aerial parts	20.4	306.7 ± 42.4	115.8 ± 13.6
8	*Artemisia princeps* Pamp. Hara (AP)	Sook	Aerial parts	16.6	915.2 ± 25.5	188.0 ± 3.9
9	*Oenanthe javanica (*blume*) DC. (*OJ)	Dolminari	Aerial parts	13.8	2292.6 ± 32.3	230.6 ± 5.0
10	*Glebionis coronaria* (GC)	Sookgod	Aerial parts	31.0	2549.3 ± 140.4	263.6 ± 11.8

(1) The yield of 50% ethanol extract from dried sample, (2) 2,2-diphenyl-1 picrylhydrazyl, (3) ferric reducing antioxidant power, (4) concentration of each extract to reduce the oxidant levels by 50%, (5) concentration of each extract to exhibit the equal effect to 10 *µ*g/mL of ascorbic acid, (6) mean ± SD.

**Table 3:** Body weight gain, diet intake, and visceral adipose tissue weight of mice.

	LFC (*n* = 7)	HFC (*n* = 8)	LY (*n* = 8)	CS-L (*n* = 8)	CS-H (*n* = 8)	OB-L (*n* = 8)	OB-H (*n* = 8)
Initial body weight (g)	21.5 ± 0.9^ns^	21.6 ± 0.5	21.8 ± 0.8	21.6 ± 0.9	21.6 ± 0.8	21.7 ± 0.5	21.5 ± 0.6
Body weight gain (g)	8.1 ± 1.5^c2)^	18.4 ± 2.7^a^	13.1 ± 2.8^b^	13.3 ± 1.9^b^	10.4 ± 1.7^c^	10.2 ± 2.3^c^	9.8 ± 2.7^c^
Diet intake (g)	270.9 ± 5.0^a^	237.0 ± 1.2^b^	237.2 ± 13.5^b^	249.3 ± 18.4^b^	235.5 ± 14.4^b^	238.1 ± 24.8^b^	235.6 ± 14.2^b^
Energy intake (kcal)	1034.7 ± 19.3^b^	1114.1 ± 5.6^a^	1114.7 ± 63.3^a^	1171.7 ± 86.5^a^	1107.0 ± 67.9^ab^	1119.1 ± 116.3^a^	1107.4 ± 66.7^ab^
Food efficient ratio (%g/g)^1)^	2.98 ± 0.55^e^	7.77 ± 1.10^a^	5.53 ± 1.11^b^	5.34 ± 0.74^bc^	4.46 ± 0.85^cd^	4.24 ± 0.58^d^	4.16 ± 1.06^d^
Epididymal fat (g)	0.73 ± 0.25^d^	2.25 ± 0.30^a^	1.62 ± 0.25^b^	1.38 ± 0.42^bc^	1.11 ± 0.42^c^	1.24 ± 0.30^c^	1.10 ± 0.21^c^
Perirenal fat (g)	0.25 ± 0.08^d^	0.96 ± 0.14^a^	0.77 ± 0.17^b^	0.58 ± 0.23^c^	0.49 ± 0.24^c^	0.56 ± 0.11^c^	0.52 ± 0.13^c^
Liver weight (g)	1.39 ± 0.21^a^	1.31 ± 0.24^ab^	1.15 ± 0.10^bc^	1.23 ± 0.17^abc^	1.16 ± 0.15^bc^	1.09 ± 0.14^c^	1.09 ± 0.12^c^

LFC: low-fat diet (LFD) control, HFC: high-fat diet (HFD) control, LY : HFD + oral treatment with lycopene beadlets at 50 mg/kg body weight (BW) containing 5% tomato lycopene (positive control), CS-L : HFD + oral treatment with 50% ethanol extract of *Cedrela sinensis* (CS) at 50 mg/kg BW, CS-H : HFD + oral treatment with CS extract 100 mg/kg BW, OB-L : HFD + oral treatment with 50% ethanol extract of *Oenothera biennis* L. (OB) at 50 mg/kg BW, OB-H : HFD + oral treatment with OB extract 100 mg/kg BW. Values are mean ± SD. ns: not significant. (1) Food efficient ratio = (body weight gain (g)/food intake (g)) x 100, (2) means sharing the same alphabet in superscripts are not significantly different within a row at *P* < 0.05 as determined by ANOVA and Duncan's multiple range test.

**Table 4:** Correlations between parameters measured in mice.

	Body weight gain		Adipocyte size	
	*r* ^1)^	Significance	*r* ^1)^	Significance
Adipocyte size	0.8110	^*∗∗∗*^	1	
*Plasma*				
MDA^2)^	0.3692	^*∗*^	0.1406	ns
8-epi-PGF2*α*^3)^	0.4358	^*∗∗*^	0.6477	^*∗∗*^
Leptin	0.7263	^*∗∗∗*^	0.7599	^*∗∗∗*^
Triglyceride	0.3767	^*∗*^	0.2269	ns
T. cholesterol	0.4913	^*∗∗*^	0.7076	^*∗∗∗*^

*Adipose tissue*				
Epididymal fat	0.8472	^*∗∗∗*^	0.8876	^*∗∗∗*^
Adipocyte size	0.8110	^*∗∗∗*^	1	
*mRNA*				
Leptin	0.8732	^*∗∗∗*^	0.7517	^*∗∗∗*^
Adiponectin	0.3160	ns	−0.3531	ns
HO-1^4)^	0.7798	^*∗∗∗*^	0.5650	^*∗∗*^
p40phox^5)^	0.7412	^*∗∗∗*^	0.4631	^*∗*^
GSTA4^6)^	0.3612	^*∗*^	−0.4899	^*∗∗*^
TNF-*α*^7)^	0.7324	^*∗∗∗*^	0.5163	^*∗∗*^
MCP-1^8)^	0.5272	^*∗∗*^	0.3541	ns
CD11c^9)^	0.8265	^*∗∗∗*^	0.7431	^*∗∗∗*^
IL-6^10)^	0.4174	^*∗*^	0.3214	ns

*Liver*				
TBARS^11)^	0.4019	^*∗∗*^	0.3395	ns
*Activity*				
Catalase	−0.3823	^*∗∗*^	−0.5647	^*∗∗∗*^
SOD-1^12)^	−0.2011	ns	−0.3858	^*∗*^
GST	−0.3115	^*∗*^	−0.5384	^*∗∗∗*^
*mRNA*				
IL-6	0.5208	^*∗∗*^	0.3566	ns
TNF-*α*	0.3407	^*∗*^	0.3078	ns

(1) Pearson correlation coefficient, (2) malondialdehyde, (3) 8-epi-prostaglandin F_2_*α*, (4) heme oxygenase-1, (5) subunit of NADPH oxidase, (6) glutathione S-transferase A4, (7) tumor necrosis factor, (8) macrophage attractant protein-1, (9) a surface marker of M1 type of macrophages, (10) interleukin-6, (11) thiobarbituric acid-reactive substance, (12) superoxide dismutase-1, significant at ^*∗*^*P* < 0.05, ^*∗∗*^*P* < 0.01, or ^*∗∗∗*^*P* < 0.001 as determined by Pearson correlation test, ns: not significant.

## Data Availability

The experimental data used to support the findings of this study are available from the corresponding author upon request.
